# Design and Synthesis of Some Thiazolidin-4-ones Based on (7-Hydroxy-2-oxo-2*H*-chromen-4-yl) Acetic Acid

**DOI:** 10.3390/molecules14072501

**Published:** 2009-07-10

**Authors:** Milan Cacic, Maja Molnar, Tomislav Balic, Nela Draca, Valentina Rajkovic

**Affiliations:** 1 Department of Applied Chemistry and Ecology, Faculty of Food Technology, J. J. Strossmayer University, Franje Kuhaca 20, 31 000 Osijek, Croatia; 2 Department of Chemistry, J. J. Strossmayer University, Franje Kuhaca 20, 31 000 Osijek, Croatia; 3 Department of Biology, J. J. Strossmayer University, Trg Ljudevita Gaja 6, 31 000 Osijek, Croatia

**Keywords:** coumarin, dihydrazides, aromatic Schiff’s bases, *N*-(2-aryl-4-oxo-thiazolidin-3-yl)-2-((4-(2-aryl-4-oxo-thiazolidin-3-ylcarbamoyl)-methyl)-2-oxo-2*H*-chromen-7-yl-oxy)-acetamides, biological activity

## Abstract

(7-Hydroxy-2-oxo-2*H*-chromen-4-yl)-acetic acid methyl ester (**1**) upon reaction with ethyl bromoacetate furnishes (7-ethoxycarbonylmethoxy-2-oxo-2*H*-chromen-4-yl)-acetic acid methylester (**2**), which on treatment with 100% hydrazine hydrate yields (7-hydrazinocarbonylmethoxy-2-oxo-2*H*-chromen-4-yl)-acetic acid hydrazide (**3**). The condensation of compound **3** with different aromatic aldehydes afforded a series of [7-(arylidenehydrazinocarbonylmethoxy)-2-oxo-2*H*-chromen-4-yl]-acetic acid arylidene-hydrazide Schiff’s bases **4a-k.** Cyclo-condensation of compounds **4a-k** with 2-mercapto-acetic acid in *N,N*-dimethylformamide in the presence of anhydrous ZnCl_2_ affords *N*-(2-aryl-4-oxothiazolidin-3-yl)-2-(4-(2-aryl-4-oxothiazolidin-3-ylcarbamoyl)-methyl)-2-oxo-2*H*-chromen-7-yloxy)-acetamides **5a-k**. Structure elucidation of the products has been accomplished on the basis of elemental analysis, IR, ^1^H-NMR and ^13^C-NMR data. Compounds **4a-k** and **5a-k** will be screened for their antibacterial activity against both Gram-positive and Gram-negative bacteria and the results reported elsewhere in due course.

## Introduction

Coumarin (2-oxo-2*H*-chromene) and its derivatives represent one of the most active classes of heterocyclic compounds, possessing a wide spectrum of biological activities [1,2,3,4,5,6,7,8,9] as many of these compounds posses antitumor [1,2], antibacterial [3,4], antifungal [5,6,7], anticoagulant [8] and anti-inflammatory [9] properties. They have also shown to be useful as anti-HIV agents and as CNS active compounds [10]. In addition, these compounds are used as additives to food and cosmetics [11], dispersed fluorescence and lasers [12]. Coumarins are present in remarkable amounts in plants, although their presence has also been detected in microorganisms and animal sources [10].

Thiazolidinones are derivatives of thiazolidine and they also constitute an important group of heterocyclic compounds. Thiazolidinones, with a carbonyl group in positions 2,4- or just 4-, have been subject of extensive study in the recent past [13,14] and literature surveys show that thiazolidin-4-ones are important compounds due to their broad range of biological activities [15,16,17,18,19,20,21]. 4-Thiazolidinones substituted in the 2-position, its derivatives and analogues exhibit unusually high *in vitro* activity against *Mycobacterium tuberculosis* [22]. Overviews of their synthesis, properties, reactions and applications have been published [13,14].

Thiazolidine compounds are formed by condensation of either aliphatic or aromatic moieties, containing a formyl group (-CHO), with different aminothiols [23]. It should be noted that the thiazolidine ring is the core building unit of penicillin antibiotics. A novel synthesis of thiazolidine-2-thione and thiazolidine-2-one derivatives is described with the iodo-cyclothiocarbamation reaction as the key step for the heterocyclic ring formation. The new method has been applied to the synthesis of thiazolidinones as bioisosteric analog of Linezolid [24]. In recent years several new methods for preparing thiazolidinone derivatives have been reported in the literature [25,26].

**Scheme 1:**
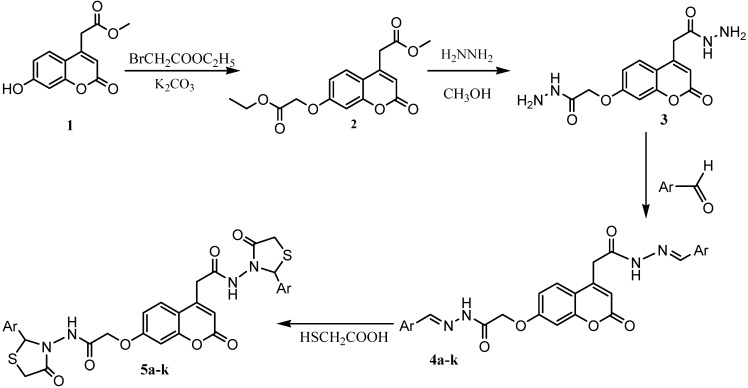
Synthetic route for thiazolidinones **5a-k.**

As part of our aim in search of biologically active heterocyclic compounds with one, two or three coumarin cores and thiazolidinone moieties, we have previously reported the synthesis of some of these compounds [27,28]*.* Since 4-thiazolidinones show a wide range of biological activities [15,16,17,18,19,20,21], we extended our work on the synthesis of novel compounds formed by cyclocondensation from compounds (Schiff’s bases) **4a-k** and mercaptoacetic acid in *N,N*-dimethylforamide in the presence of anhydrous ZnCl_2_, to afford *N*-(2-aryl-4-oxothiazolidin-3-yl)-2-(4-(2-aryl-4-oxothiazolidin-3-yl-carbamoyl)-methyl)-2-oxo-2*H*-chromen-7-yloxy)-acetamides **5a-k** (Scheme 1).

Thiazolidinone coumarin derivatives have been proven to have significant biological activity, like anticonvulsant activity [29], cytotoxic activity [30] and antioxidant activity [31]. Therefore, our aim in this work was to prepare thiazolidinone derivatives using (7-hydroxy-2-oxo-2*H*-chromen-4-yl)-acetic acid as starting compound.

## Results and Discussion

### Synthesis

The synthesis of the target compounds was carried out as outlined in Scheme 1. The starting compound (7-hydroxy-2-oxo-2*H*-chromen-4-yl)-acetic acid methyl ester (**1**) was prepared in 92% yield by esterification of (7-hydroxy-2-oxo-2*H*-chromen-4-yl)-acetic acid [28]. We have previously reported the preparation of (7-ethoxycarbonylmethoxy-2-oxo-2*H*-chromen-4-yl)-acetic acid methyl ester (**2**) in 82% yield by direct condensation of **1** with bromoacetic acid ethyl ester [28].

**Table:** 

Entry	Ar	Entry	Ar
**a**	Phenyl	**g**	4-Hydroxy-3-methoxyphenyl
**b**	2-Chlorophenyl	**h**	3-Phenoxyphenyl
**c**	3-Chlorophenyl	**i**	4- *N,N*-Dimethylaminophenyl
**d**	2,4-Dihydroxyphenyl	**j**	2-Hydroxy-5-nitrophenyl
**e**	3,4-Dihydroxyphenyl	**k**	Styryl
**f**	2,5-Dihydroxyphenyl		

Hydrazinolysis of **2** with 86% hydrazine hydrate in methanol at room temperature afforded dihydrazide **3** in good yield. The FT-IR spectra of carbohydrazide **3** showed absorption bands in the 3,317 cm^-1 ^(hydrazide NH-NH_2_), 3,269 cm^-1^(aromatic C-H), 1,711 cm^-1^(-C=O carbonyl stretching) and 1,621-1,640 cm^-1^(-CO-NH-NH_2_ groups) regions, respectively. The ^1^H-NMR spectrum exhibited a singlet due to the –CO-NH-NH_2_ NH proton at δ 9.32 ppm. Methylene protons –CH_2_ and –OCH_2_ resonated as singlets at 4.23 and 4.85 ppm, respectively.

A new series of compounds **4a-k** was prepared similarly to those previously described [28] by refluxing a solution of suitable different aromatic aldehydes and dihydrazide **3** in absolute ethanol for 2 to 4 hours, in a presence of a catalytic amount of glacial acetic acid. The structures of compounds **4a-k** were inferred from their analytical and spectral data.

Thus, their IR spectra showed characteristic bands at 3,448-3,278 cm^-1^(NH), 1,709 cm^-1^, 1,672 cm^-1 ^(C=O, lactone) and 1,620 cm^-1^(C=O, amide, -HC=N- azomethine). The ^1^H-NMR spectra did not only show the absence of NH_2_ protons at 3.38, but also the presence of the N=CH proton at 8.30 ppm.

N-(2-aryl-4-oxo-thiazolidin-3-yl)-2-(4-(2-aryl-4-oxo-thiazolidin-3-ylcarbamoyl)-methyl)-2-oxo-2H-chromen-7-yloxy)-acetamides **5a-k** were obtained by refluxing a solution of compounds **4a-k** and thioglicolic acid in *N,N*-dimethylformamide for 6-8 hours in the presence of anhydrous ZnCl_2_.

## Experimental

### General

Melting points were determined on Electrothermal Capillary melting point apparatus and are uncorrected. Thin-layer chromatography was performed with fluorescent silica gel plates HF_254_ Merck, which were checked under UV 254 and 365 nm light. The elemental analysis for C, H and N was done on a Perkin-Elmer Analyzer 2440. Infrared spectra (ν_max_-cm^-1^) were recorded on a Beckmann FT-IR 3303, using KBr disks. ^1^H-NMR spectra were recorded on JEOL EX-270 MHz NMR Spectrometer at 293 K in DMSO-d_6_. ^13^C-NMR spectra were recorded on a Varian Gemini at 50 MHz in DMSO-d_6_. Spectra were internally referenced to TMS. Peaks are reported in ppm downfield of TMS.

### (7-Ethoxycarbonylmethoxy-2-oxo-2H-chromen-4-yl)-acetic acid methylester (**2**)

A mixture of (7-hydroxy-2-oxo-2*H*-chromen-4-yl)-acetic acid methylester (**1,** 25.74 g, 0.11 mole), anhydrous potassium carbonate (15.20 g, 0.11 mole) and ethyl bromoacetate (18.37 g, 0.11 mole) in dry acetone (200 mL) was refluxed with continuous stirring for 12 hours. After filtration, the solution was concentrated under reduced pressure, vacuum dried and the solid product was recrystallized from ethanol.M.p.185-186^◦^C, yield 64%; IR: ν_max_ 3,429, 2,986, 2,941, 1,753, 1,724, 1,619, 1,439, 1,393, 1,341, 1,221, 1,198, 1,089 cm^-1^; ^1^H-NMR: δ 7.76 (d, 1H, H-5), 7.04 (d, 1H, H-6), 7.02 (s, 1H, H-8), 6.34 (s, 1H, H-3), 4.92 (s, 2H, -OCH_2_), 4.19 (q, 2H, CH_2_, -CH_2_CH_3_), 4.02 (s, 2H, CH_2_), 3.65 (s, 3H, OCH_3_), 1.22 (t, 3H, CH_3_, -CH_2_CH_3_); ^13^C-NMR: δ 14.2 (CH_2_CH_3_), 34.8 (CH_2_CO), 52.1 (OCH_3_), 61.3 (CH_2_CH_3_), 65.5 (COCH_2_O), 109.6 (C-8), 112.8 (C-6), 113.8 (C-3), 114.8 (C-10), 128.3 (C-5), 151.2 (C-9), 155.2 (C-4), 160.3 (C-7), 160.9 (C-2), 168.9 (CO-O), 169.3 (C-CO-C); Anal. Calcd. for C_16_H_16_O_7_: C, 60.00; H, 5.04; Found: C, 59.98; H, 5.01%.

### (7-Hydrazinocarbonylmethoxy-2-oxo-2H-chromen-4-yl)-acetic acid hydrazide (**3**)

To a solution of methanol (120 mL) and 86% hydrazine hydrate (12 mL) (7-ethoxycarbonylmethoxy-2-oxo-2*H*-chromen-4-yl)-acetic acid methylester (**2**, 3.2 g, 0.01 mole) was added, and the mixture was left to stand overnight at 5^◦^C. The product precipitated and was collected by suction filtration, washed with methanol (petrolether) and recrystallized from dil. acetic acid. M.p.> 300^◦^C, yield 70%; IR: ν_max_ 3,461, 3,325 (NH), (NH_2_), 1,707 (lactone C=O), 1,623 (C=O, amide), 1,516 (C=C, arom.), 1,430, 1,298, 1,277 and 1,153 cm^-1^; ^1^H-NMR: δ 9.41 (s, 1H, NH), 9.34 (s, 1H, NH), 7.76 (d, 1H, H-5), 7.04 (d, 1H, H-6), 7.02 (s, 1H, H-8), 6.34 (s, 1H, H-3), 4.94 (s, 2H, -OCH_2_), 4.34 (s, 2H, NH_2_), 4.08 (s, 2H, CH_2_), 3.38 (s, 2H, NH_2_); ^13^C-NMR: δ 45.8 (CH_2_), 68.9 (CH_2_O-), 108.0 (C-8), 111.8 (C-6), 112.9 (C-3), 114.1 (C-10), 128.3 (C-5), 152.2 (C-9), 155.2 (C-4), 160.4 (C-7), 160.9 (C-2), 166.8 (COCH_2_O), 169.6 (COCH_2_); Anal. Calcd. for C_13_H_14_N_4_O_5_: C, 50.98; H, 4.61; N, 18.29; Found: C, 51.02; H, 4.58; N, 18.25%.

### General procedure for preparation of (7-(arylidene-hydrazinocarbonylmethoxy)-2-oxo-2H-chromen-4-yl)-acetic acid aryilidene-hydrazides **4a-k**

A mixture of (7-Hydrazinocarbonylmethoxy-2-oxo-2H-chromen-4-yl)-acetic acid hydrazide (**3,** 3.06 g, 0.01 mole) and appropriate aromatic aldehyde (**Ar/a-k**, 0.01 mole) was refluxed in absolute ethanol (30 mL) in the presence of a catalytic amount of glacial acetic for 2 to 4 hours. The reaction mixture was cooled, the solid separated was filtered and recrystallized from methanol to give compounds **4a-k**.

*[7-(Benzylidene-hydrazinocarbonylmethoxy)-2-oxo-2H-chromen-4-yl]-acetic acid benzylidene hydrazide* (**4a**). M.p. 268-269^◦^C; yield 74%; IR: ν_max_ 3,418, 3,313 (NH), 1,712, 1,682 (C=O, lactone), 1,666 (C=O, amide), 1,613 (C=C, arom., C=N, azomet.), 1,550, 1,378, 1,269 and 1,153 cm^-1^; ^1^H- NMR: δ 8.30 (s, 1H, HC=N-), 8.24 (s, 1H, HC=N-), 8.06 (s, 1H, NH), 8.02 (s, 1H, NH), 7.76 (d, 1H, H-5), 7.72-7.31 (m, 10H, arom.), 7.04 (d, 1H, H-6), 7.02 (s, 1H, H-8), 6.34 (s, 1H, H-3), 4.83 (s, 2H, -OCH_2_), 4.28 (s,2H,CH_2_); ^13^C-NMR: δ 45.7 (CH_2_), 69.2 (CH_2_O-), 108.1 (C-8), 111.6 (C-6), 112.5 (C-3), 114.4 (C-10), 127.9 (C-5), 128.4 (C-3,5, Ar-), 129.0 (C-2,6, Ar-), 131.4 (C-4, Ar-), 133.9 (C-1, Ar-), 143.6 (N=CH-), 151.8 (C-9), 155.2 (C-4), 160.4 (C-7), 160.9 (C-2), 166.9 (COCH_2_O), 170.0 (CONH-); Anal. Calcd. for C_27_H_22_N_4_O_5_: C, 67.21; H, 4.60; N, 11.61; Found: C, 67.19; H, 4.61; N, 11.58%.

*[7-(2-Chlorobenzylidenehydrazinocarbonylmethoxy)-2-oxo-2H-chromen-4-yl]-acetic acid (2-chloro-benzylidene)- hydrazide* (**4b**). M.p. 225-226^◦^C, yield (76%); IR: ν_max_ 3,428, 3,283 (NH), 1,710, 1,692 (C=O, lactone), 1,656 (C=O, amide), 1,612 (C=C, arom., C=N, azomet.), 1,542, 1,398, 1,264 and 1,155 cm^-1^; ^1^H-NMR: δ 8.73 (s, 1H, -HC=N-), δ 8.62 (s, 1H, -HC=N-), δ 8.48 (s, 1H, NH), 8.45 (s, 1H, NH), 7.72 (d, 1H, H-5), 7.60-7.30 (m, 8H, arom.), 7.04 (d, 1H, H-6), 7.02 (s, 1H, H-8), 6.34 (s, 1H, H-3), 4.84 (s, 2H, -OCH_2_), 4.30 (s, 2H, CH_2_); ^13^C-NMR: δ 45.6 (CH_2_), 68.9 (CH_2_O-), 107.8 (C-8), 111.5 (C-6), 112.7 (C-3), 113.9 (C-10), 127.8 (C-5), 129.4 (C-3, Ar-), 130.6 (C-6, Ar-), 132.7 (C-4, Ar-), 133.8 (C-1, Ar-), 134.3 (C-2, Ar-), 143.5 (N=CH-), 151.7 (C-9), 155.0 (C-4), 160.3 (C-7), 160.9 (C-2), 166.4 (COCH_2_O), 169.9 (CONH-); Anal. Calcd. For C_27_H_20_Cl_2_N_4_O_5_: C, 58.81; H, 3.66; N, 10.16; Found: C, 58.79; H, 3.69; N, 10.12%.

*[7-(3-Chlorobenzylidene-hydrazinocarbonylmethoxy)-2-oxo-2H-chromen-4-yl]-acetic acid (3-chloro-benzylidene)-hydrazide* (**4c**). M.p. 259-261^◦^C, yield 72%; IR: ν_max_ 3,408, 3,188 (NH), 1,727, 1,683 (C=O, lactone), 1,616 (C=O, amide, C=N, azomet.), 1,561 (C=C, arom.), 1,394, 1,262 and 1,138 cm^-1^; ^1^H-NMR: δ 8.31 (s, 1H, -HC=N-), 8.22 (s, 1H, -HC=N-), 8.48 (s, 1H, NH), 8.45 (s, 1H, NH), 7.76 (d, 1H, H-5), 7.67-7.30 (m, 8H, arom.), 7.04 (d, 1H, H-6), 7.02 (s, 1H, H-8), 6.34 (s, 1H, H-3), 4.84 (s, 2H, -OCH_2_), 4.28 (s, 2H, CH_2_); ^13^C-NMR: δ 45.5 (CH_2_), 69.1 (CH_2_O-), 107.9 (C-8), 111.4 (C-6), 112.5 (C-3), 113.4 (C-10), 127.3 (C-6, Ar-), 127.9 (C-5), 129.3 (C-2, Ar-), 130.3 (C-5, Ar-), 131.2 (C-4, Ar-), 135.2 (C-1, Ar-), 134.4 (C-3, Ar-), 143.4 (N=CH-), 151.6 (C-9), 155.2 (C-4), 160.6 (C-7), 160.9 (C-2), 166.7 (COCH_2_O), 169.8 (CONH-); Anal. Calcd. For C_27_H_20_Cl_2_N_4_O_5_: C, 58.81; H, 3.66; N, 10.16; Found: C, 58.80; H, 3.68; N, 10.13%.

*[7-(2,4-Dihydroxy-benzylidene-hydrazinocarbonylmethoxy)-2-oxo-2H-chromen-4-yl]-acetic acid (2,4-dihydroxybenzylidene)-hydrazide* (**4d**). M.p. 273-275^◦^C, yield 52%; IR: ν_max_ 3,434 (OH), 3,366, 3,092 (NH), 1,712, 1,672 (C=O, lactone), 1,623 (C=O, amide, C=N, azomethine), 1,612 (C=C, arom., C=N), 1,559, 1,509, 1,395, 1,265 and 1,153 cm^-1^; ^1^H-NMR: δ 11.80 (s, 1H, OH), 11.17 (s, 1H, OH), 8.42 (s, 1H, -HC=N-), 8.30 (s, 1H, -HC=N-), 8.23 (s, 1H, NH), 8.19 (s, 1H, NH), 7.76 (d, 1H, H-5), 7.61-7.30 (m, 6H, arom.), 7.04 (d, 1H, H-6), 7.02 (s, 1H, H-8), 6.34 (s, 1H, H-3), 4.82 (s, 2H, -OCH_2_), 4.28 (s, 2H, CH_2_); ^13^C-NMR: δ 45.6 (CH_2_), 69.2 (CH_2_O-), 103.8 (C-3, Ar-), 107.6 (C-8), 108.7 (C-5, Ar-), 111.3 (C-6), 112.7 (C-3), 113.4 (C-10), 127.2 (C-6, Ar-), 127.9 (C-5), 135.2 (C-1, Ar-), 143.0 (N=CH-), 151.2 (C-9), 155.1 (C-4), 160.5 (C-7), 160.9 (C-2), 162.4 (C-2, Ar-), 162.6 (C-4, Ar-), 166.5 (COCH_2_O), 169.4 (CONH-); Anal. Calcd. For C_27_H_22_N_4_O_9_: C, 59.34; H, 4.06; N, 10.25; Found: C, 59.30; H, 4.07; N, 10.29%.

*[7-(3,4-Dihydroxy-benzylidene-hydrazinocarbonylmethoxy)-2-oxo-2H-chromen-4-yl]- acetic acid (3,4-dihydroxybenzylidene)-hydrazide* (**4e**). M.p. 205^◦^C, yield 62%; IR: ν_max_ 3,408, 2,922 (NH), 1,725, 1,664 (C=O, lactone), 1,619 (C=O, amide, C=N, azomethine), 1,593 (C=C, arom.), 1,444, 1,393, 1,284 and 1,152 cm^-1^; ^1^H-NMR: δ 11.98 (s, 1H, OH), 11.45 (s, 1H, OH), 8.41 (s, 1H, -HC=N-), 8.30 (s, 1H, -HC=N-), 8.12 (s, 1H, NH), 8.03 (s, 1H, NH), 7.76 (d, 1H, H-5), 7.65-7.41 (m, 6H, arom.), 7.04 (d, 1H, H-6), 7.02 (s, 1H, H-8), 6.34 (s, 1H, H-3), 4.77 (s, 2H, -OCH_2_), 4.22 (s, 2H, CH_2_); ^13^C-NMR: δ 45.5 (CH_2_), 69.3 (CH_2_O-), 107.6 (C-8), 111.4 (C-6), 112.5 (C-3), 113.5 (C-10), 116.4 (C-2, Ar-), 117.5 (C-5, Ar-), 123.3 (C-6, Ar-), 127.8 (C-5), 127.9 (C-1, Ar-), 143.1 (N=CH-), 147.4 (C-3, Ar-), 149.6 (C-4, Ar-), 151.3 (C-9), 155.0 (C-4), 160.4 (C-7), 160.9 (C-2), 166.6 (COCH_2_O), 169.5 (CONH-); Anal. Calcd. For C_27_H_22_N_4_O_9_: C, 59.34; H, 4.06; N, 10.25; Found: C, 59.13; H, 4.03; N, 10.04%.

*[7-(2,5-Dihydroxybenzylidene-hydrazinocarbonylmethoxy)-2-oxo-2H-chromen-4-yl]- acetic acid (2,5-dihydroxybenzylidene)-hydrazide* (**4f**). M.p. 275-276^◦^C, yield 76%; IR: ν_max_ 3,369, 3,286 (NH), 1,717, 1,681, 1,667 (C=O, lactone), 1,624 (C=O, amide, C=N, azomethine), 1,585 (C=C arom.), 1,492, 1,396, 1,267 and 1,156 cm^-1^; ^1^H-NMR: δ 11.95 (s, 1H,OH), 11.56 (s,1H,OH), 8.48 (s, 1H, -HC=N-), 8.34 (s, 1H, -HC=N-), 8.30 (s, 1H, NH), 8.25 (s, 1H, NH), 7.76 (d, 1H, H-5), 7.68-7.30 (m, 6H, arom.), 7.04 (d, 1H, H-6), 7.02 (s, 1H, H-8), 6.34 (s, 1H, H-3), 4.82 (s, 2H, -OCH_2_), 4.24 (s, 2H, CH_2_); ^13^C-NMR: δ 45.6 (CH_2_), 69.3 (CH_2_O-), 107.8 (C-8), 111.4 (C-6), 112.7 (C-3), 113.8 (C-10), 116.4 (C-6, Ar-), 117.4 (C-3, Ar-), 119.6 (C-4, Ar-), 119.9 (C-1, Ar-), 127.8 (C-5), 143.5 (N=CH-), 151.4 (C-9), 151.3 (C-5, Ar-), 153.7 (C-2, Ar-), 155.2 (C-4), 160.4 (C-7), 160.9 (C-2), 166.7 (COCH_2_O), 169.4 (CONH-); Anal. Calcd. For C_27_H_22_N_4_O_9_: C, 59.34; H, 4.06; N, 10.25; Found: C, 59.32; H, 4.04; N, 10.20%.

*[7-(4-Hydroxy-3-methoxybenzylidene-hydrazinocarbonylmethoxy)-2-oxo-2H-chromen-4yl]-acetic acid(4-hydroxy-3-methoxybenzylidene)-hydrazide* (**4g**). M.p. 232-233^◦^C, yield 84%; IR: ν_max_ 3,430, 3,224 (NH), 1,711, 1,671 (C=O, lactone), 1,622 (C=O, amide, C=N, azomethine), 1,605 (C=C, arom.), 1,429, 1,394, 1,272 and 1,164 cm^-1^; ^1^H-NMR: δ 11.96 (s, 1H, OH), δ 11.50 (s, 1H, OH), 8.19 (s, 1H, -HC=N-), 8.10 (s, 1H, -HC=N-), 7.99 (s, 1H, NH), 7.97 (s, 1H, NH), 7.77 (d, 1H, H-5), 7.40-7.21 (m, 6H, arom.), 7.04 (d, 1H, H-6), 7.02 (s, 1H, H-8), 6.34 (s, 1H, H-3), 4.78 (s, 2H, -OCH_2_), 4.24 (s, 2H, CH_2_), 3.80 (s, 6H, -OCH_3_); ^13^C-NMR: δ 45.6 (CH_2_), 56.0 (OCH_3_), 69.2 (CH_2_O-), 107.6 (C-8), 111.2 (C-6), 112.5 (C-3), 113.6( C-10), 114.8 (C-2, Ar-), 117.0 (C-5, Ar-), 122.9 (C-6, Ar-), 127.4 (C-1,Ar-), 127.8 (C-5), 143.3 (N=CH-), 148.1 (C-4, Ar-), 151.4 (C-9), 151.5 (C-3, Ar-), 155.1 (C-4), 160.4 (C-7), 160.9 (C-2), 166.8 (COCH_2_O), 169.8 (CONH-); Anal. Calcd. For C_29_H_26_N_4_O_9_: C, 60.62; H, 4.56; N, 9.75; Found: C, 60.59; H, 4.75; N, 9.70%.

*[7-(3-Phenoxybenzylidene-hydrazinocarbonylmethoxy)-2-oxo-2H-chromen-4-yl]-acetic acid 3-phenoxybenzylidene)-hydrazide* (**4h**). M.p. 236-237^◦^C, yield 57%; IR: ν_max_ 3,409, 3,071 (NH), 1,726, 1,685 (C=O, lactone), 1,624 (C=O, lactone, C=N, azomethine), 1,597 (C=C, arom.), 1,490, 1,394, 1,261 and 1,156 cm^-1^; ^1^H-NMR: δ 8.30 (s, 1H, -HC=N-), 8.21 (s, 1H, -HC=N-), 8.03 (s, 1H, NH), 7.99 (s, 1H, NH), 7.76 (d, 1H, H-5), 7.70-7.10 (m, 18H, arom.), 7.04 (d, 1H, H-6), 7.02 (s, 1H, H-8), 6.34 (s, 1H, H-3), 4.79 (s, 2H, -OCH_2_), 4.18 (s, 2H, CH_2_); ^13^C-NMR: δ 45.5 (CH_2_), 56.1 (OCH_3_), 69.1 (CH_2_O-), 107.8 (C-8), 111.4 (C-6), 112.7 (C-3), 113.5 (C-10), 116.6 (C-2, Ar-), 117.5 (C-2,6, Ar-PhO), 119.8 (C-4, Ar-), 121.9 (C-4, Ar- PhO), 122.3 (C-2, Ar-), 127.8 (C-5), 128.5 (C-3,5 Ar- PhO), 128.9 (C-5 Ar-), 133.5 (C-1 Ar-), 143.4 (N=CH-), 151.3 (C-9), 155.4 (C-4), 157.1 (C-1 Ar- PhO), 157.1 (C-3 Ar-), 160.4 (C-7), 160.9 (C-2), 166.8 (COCH_2_O), 170.0 (CONH-); Anal. Calcd. For C_39_H_30_N_4_O_7_: C, 70.26; H, 4.54; N, 8.40; Found: C, 70.23; H, 4.55; N, 8.37%.

*[7-(4-N,N-Dimethylaminobenzylidene-hydrazinocarbonylmethoxy)-2-oxo-2H-chromen-4-yl]-acetic acid (4-N,N-dimethylaminobenzylidene)-hydrazide* (**4i**). M.p. 207-209^◦^C, yield 63%; IR: ν_max _3,408, 3,082 (NH), 1,724, 1,679 (C=O, lactone), 1,623 (C=O, amide, C=N, azomethine), 1,604 (C=C, arom.), 1,554, 1,525, 1,364, 1,269 and 1,181cm^-1^; ^1^H-NMR: δ 8.49 (s, 1H, -HC=N-), 8.44 (s, 1H, -HC=N-), 8.17 (s, 1H, NH), 8.07 (s, 1H, NH), 7.66 (d, 1H, H-5), 7.24-7.52 (m, 8H, arom.), 7.04 (d, 1H, H-6), 7.02 (s, 1H, H-8), 6.34 (s, 1H, H-3), 4.74 (s, 2H, -OCH_2_), 4.18 (s, 2H, CH_2_), 3.32 (s, 6H, -N(CH_3_)_2_), 2.99 (s, 6H, -N(CH_3_)_2_); ^13^C-NMR: δ 40.3 (CH_3_N-), 45.6 (CH_2_), 69.1 (CH_2_O-), 107.6 (C-8), 111.3 (C-6), 112.7 (C-3), 113.8 (C-10), 114.4 (C-3,5 Ar-), 123.3 (C-1, Ar-), 127.8 (C-5), 130.2 (C-2,6 Ar-), 143.3 (N=CH-), 151.4 (C-9), 151.0 (C-4, Ar-), 155.5 (C-4), 160.6 (C-7), 160.9 (C-2), 166.7 (COCH_2_O), 169.8 (CONH-); Anal. Calcd. For C_31_H_32_N_6_O_5_: C, 64.97; H, 5.45; N, 15.15; Found: C, 65.89; H, 5.58; N, 15.11 %.

*[7-(2-Hydroxy-5-nitrobenzylidenehydrazinocarbonylmethoxy)-2-oxo-2H-chromen-4-yl]-acetic acid (2-hydroxy-5-nitrobenzylidene)-hydrazide* (**4j**). M.p. 204^◦^C, yield 82%; IR: ν_max_ 3,367, 3,272 (NH), 1,706, 1,689 (C=O, lactone), 1,616 (C=O, C=N, azomethine), 1,600 (C=C, arom.), 1,577, 1,517, 1,481, 1,342, 1,287 and 1,150 cm^-1^; ^1^H-NMR: δ 12.02 (s, 2H, OH), 8.71 (s, 1H, -HC=N-), 8.59 (s, 1H, -HC=N-), 8.36 (s, 1H, NH), 8.31 (s, 1H, NH), 7.67 (d, 1H, H-5), 7.32-7.54 (m, 6H, arom.), 7.04 (d, 1H, H-6), 7.02 (s, 1H, H-8), 6.34 (s, 1H, H-3), 4.84 (s, 2H, -OCH_2_), 4.08 (s, 2H, CH_2_); ^13^C-NMR: δ 45.7 (CH_2_), 69.2 (CH_2_O-), 107.8 (C-8), 111.5 (C-6), 112.4 (C-3), 113.8 (C-10), 116.9 (C-3, Ar-), 119.4 (C-1, Ar-), 124.8 (C-4, Ar-), 125.5 (C-2, Ar-), 127.8 (C-5), 141.6 (C-5, Ar-), 143.4 (N=CH-), 151.4 (C-9), 155.4 (C-4), 160.8 (C-7), 160.9 (C-2), 166.2 (C-2, Ar-), 166.8 (COCH_2_O), 170.0 (CONH-); Anal. Calcd. For C_27_H_20_N_6_O_11_: C, 53.65; H, 3.33; N, 13.90; Found: C, 53.63; H, 3.35; N, 13.91%.

*[2-Oxo-7-(3-phenylallylidenehydrazinocarbonylmethoxy)-2-oxo-2H-chromen-4-yl]-acetic acid (3-phenylallylidene)-hydrazide* (**4k**). M.p. 290-292^◦^C, yield 68%; IR: ν_max_ 3,428, 3,256 (NH), 1,718 (C=O, lactone), 1,624 (C=O, amide, C=N, azomethine), 1,613 (C=C, arom.), 1,560, 1,509, 1,393, 1,266 and 1,151 cm^-1^; ^1^H-NMR: δ 8.38 (s, 1H, -HC=N-), 8.24 (s, 1H, -HC=N-), 8.15 (s, 1H, NH), 8.08 (s, 1H, NH), 7.78 (2d, 4H, -HC=CH-), 7.64 (d, 1H, H-5), 7.04 (d, 1H, H-6), 7.02 (s, 1H, H-8), 6.34 (s, 1H, H-3), 4.77 (s, 2H, -OCH_2_), 4.08 (s, 2H, CH_2_); ^13^C-NMR: δ 45.6 (CH_2_), 69.1 (CH_2_O-), 107.8 (C-8), 111.4 (C-6), 112.8 (C-3), 113.4 (C-10), 126.3 (C-2, Ar-), 126.4 (C-2,6, Ar-), 127.8 (C-5), 128.0 (C-4, Ar-), 128.9 (C-3,5, Ar-), 135.1 (C-1, Ar-), 139.0 (C-3, Ar-), 143.3 (N=CH-), 151.2 (C-9), 155.4 (C-4), 160.5 (C-7), 160.9 (C-2), 166.7 (COCH_2_O), 169.8,(CONH-); Anal. Calcd. For C_31_H_26_N_4_O_5_: C, 69.65; H, 4.90; N, 10.48; Found: C, 69.67; H, 4.88; N, 10.45%.

### General procedure for the preparation of N-(2-aryl-4-oxo-thiazolidin-3-yl)-2-(4-(2-aryl-4-oxo-thiazolidin-3-ylcarbamoyl)-methyl)-2-oxo-2H-chromen-7-yloxy)-acetamides **5a-k**

A mixture of (7-(arylidene-hydrazinocarbonylmethoxy)-2-oxo-2H-chromen-4-yl)-acetic acid aryilidenehydrazide **4a-k** (0.01 mole) and mercaptoacetic acid (1.82 g, 0.02 mole) in DMF (30 mL) containing a pinch of anhydrous ZnCl_2_ was refluxed 6-8 hours. The reaction mixture was cooled and poured onto crushed ice. The solid thus obtained was filtered, washed with water and recrystallized from DMF yielding **5a-k**.

*2-{2-Oxo-7-[(4-oxo-2-phenylthiazolidin-3-ylcarbamoyl)-methoxy]-2H-chromen-4-yl}-N-(4-oxo-2-phenylthiazolidin-3-yl)acetamide* (**5a**). M.p. 202-204^◦^C, yield 40%; IR: ν_max_ 3,418, 3,313 (NH), 1,712, 1,682 (C=O, lactone), 1,666 (C=O, amide), 1,613 (C=C, arom.), 1,550, 1,378, 1,269 and 1,153 cm^-1^; ^1^H-NMR: δ 8.22 (s, 1H, -NH), 8.12 (s, 1H, -NH), 7.76 (d,1H,H-5), 7.71-7.23 (m, 10H, arom.), 7.04 (d, 1H, H-6), 7.02 (s, 1H, H-8), 6.34 (s, 1H, H-3), 5.92 (s, 1H, -SCHN-), 4.83 (s, 2H, -OCH_2_), 4.28 (s, 2H, CH_2_), 3.38 (s, 2H, COCH_2_S-); ^ 13^C-NMR: δ 35.8 (COCH_2_S), 45.5 (CH_2_), 57.4 (NCHS), 69.1 (CH_2_O-), 107.6 (C-8), 111.0 (C-6), 112.5 (C-3), 113.4 (C-10), 127.2 (C-4, Ar-), 127.8 (C-5), 128.7 (C-3,5, Ar-), 128.8 (C-2,6 Ar-), 139.2 (C-1, Ar-), 151.2 (C-9), 155.0 (C-4), 160.3 (C-7), 160.9 (C-2), 166.4 (COCH_2_O), 168.8 (SCH_2_CO-N), 173.3 (CONH-); Anal. Calcd. For C_31_H_26_N_4_O_7_S_2_: C, 59.94; H, 4.16; N, 8.88; S, 10.17; Found: C, 60.05; H, 4.14; N, 8.91; S, 10.14%.

*N-[2-(2-Chlorophenyl)-4-oxo-thiazolidin-3-yl]-2-(7-{[2-(2-chlorophenyl)-4-oxo-thiazolidin-3-ylcarbamoyl]-methoxy}-2-oxo-2H-chromen-4-yl)-acetamide* (**5b**). M.p. 184^◦^C, yield 76%; IR: ν_max_ 3,425, 3,283 (NH), 1,692 (C=O, lactone), 1,656 (C=O, amide), 1,612 (C=C, arom.), 1,542, 1,398, 1,264 and 1,155 cm^-1^; ^1^H-NMR: δ 8.73 (s, 1H, NH-), 8.62 (s, 1H, NH-), 7.76 (d, 1H, H-5), 7.60-7.30 (m, 8H, arom.), 7.04 (d, 1H, H-6), 7.02 (s, 1H, H-8), 6.34 (s, 1H, H-3), 5.92 (s, 1H, NCHS), 4.84 (s, 2H, -OCH_2_), 4.30 (s, 2H, CH_2_), 3.38 (s, 2H, COCH_2_S); ^13^C-NMR: δ 35.7 (COCH_2_S), 45.5 (CH_2_), 57.4 (NCHS), 69.10 (CH_2_O-), 107.6 (C-8), 111.0 (C-6), 112.5 (C-3), 113.4 (C-10), 127.0 (C-5), 129.0 (C-3, Ar-), 130.6 (C-6, Ar-), 132.5 (C-4, Ar-), 133.4 (C-1, Ar-), 134.0 (C-2, Ar-), 143.0 (N=CH-), 151.2 (C-9), 155.0 (C-4), 160.3 (C-7), 160.9 (C-2), 166.4 (COCH_2_O), 173.0 (CONH-); Anal. Calcd. For C_31_H_24_Cl_2_N_4_O_7_S_2_: C, 53.22; H, 3.46; N, 8.01; S, 9.17; Found: C, 53.18; H, 3.44; N, 7.89; S, 9.20%.

*N-[2-(3-Chlorophenyl)-4-oxo-thiazolidin-3-yl]-2-(7-{[2-(3-chlorophenyl)-4-oxo-thiazolidin-3-ylcarbamoyl]-methoxy}-2-oxo-2H-chromen-4-yl)-acetamide* (**5c**). M.p. 240-241^◦^C, yield 72%; IR: ν_max_ 3,450, 3,188 (NH), 1,727, 1,683 (CO, lactone), 1,616 (C=O, amide), 1,598 (C=C, arom.), 1,394, 1,262 and 1,138 cm^-1^; ^1^H-NMR: δ 8.31 (s, 1H, NH-), 8.22,(s, 1H, NH-), 7.76 (d, 1H, H-5), 7.67-7.30 (m, 8H, arom.), 7.04 (d, 1H, H-6), 7.02 (s, 1H, H-8), 6.34 (s, 1H, H-3), 5.92 (s, 1H, NCHS), 4.84 (s, 2H, -OCH_2_), 4.28 (s, 2H, CH_2_), 3.38 (s, 2H, COCH_2_S); ^13^C-NMR: δ 35.7 (COCH_2_S), δ 45.5 (CH_2_), 57.4 (NCHS), 69.10 (CH_2_O-), 107.6 (C-8), 111.0 (C-6), 112.5 (C-3), 113.4 (C-10), 127.3 (C-6, Ar-), 127.8 (C-5), 129.3 (C-2, Ar-), 130.3 (C-5, Ar-), 131.2 (C-4, Ar-), 135.2 (C-1, Ar-), 134.4 (C-3, Ar-), 143.0 (N=CH-), 151.2 (C-9), 155.0 (C-4), 160.3 (C-7), 160.9 (C-2), 173.0 (COCH_2_O), 173.0 (CONH-); Anal. Calcd. For C_31_H_24_Cl_2_N_4_O_7_S_2_: C, 53.22; H, 3.46; N, 8.01; S, 9.17; Found: C, 53.18; H, 3.44; N, 7.89; S, 9.20%.

*N-[2-(2,4-Dihydroxyphenyl)-4-oxo-thiazolidin-3-yl]-2-(4-{[2-(2,4-dihydroxyphenyl)-4-oxo-thiazolidin-3-ylcarbamoyl]-methyl}-2-oxo-2H-chromen-7-yloxy)-acetamide* (**5d**). M.p. 239-241^◦^C, yield 52%; IR: ν_max_ 3,266 (OH), 3,092 (NH), 1,712, 1,672 (C=O, lactone), 1,624 (C=O, amide), 1,612 (C=C, arom.), 1,559, 1,509, 1,395, 1,265 and 1,153 cm^-1^; ^1^H-NMR: δ 11.80 (s, 1H, OH), 11.17 (s, 1H, OH), 8.42 (s, 1H, NH-), 8.30 (s, 1H, NH-), 7.76 (d, 1H, H-5), 7.61-7.30 (m, 6H, arom.), 7.04 (d, 1H, H-6), 7.02 (s, 1H, H-8), 6.34 (s, 1H, H-3), 5.92 (s, 1H, NCHS), 4.82 (s, 2H, -OCH_2_), 4.28 (s, 2H, CH_2_), 3.38 (s, 2H, COCH_2_S); ^13^C-NMR: δ 35.7 (COCH_2_S), δ 45.5 (CH_2_), 47.4 (NCHS), 69.10 (CH_2_O-), 103.7 (C-3, Ar-), 107.6 (C-8), 108.4 (C-5, Ar-), 110.1 (C-1, Ar-), 111.0 (C-6), 112.5 (C-3), 113.4 (C-10), 127.8 (C-5), 131.3 (C-6, Ar-), 151.2 (C-9), 157.2 (C-2, Ar-), 158.2 (C-4), 160.3 (C-7), 160.9 (C-2), 166.4 (CONH), 168.8 (NCOCH_2_), 173.3 (CH_2_CONH); Anal. Calcd. For C_31_H_26_N_4_O_11_S_2_: C, 53.60; H, 3.77; N, 8.07; S, 9.23; Found: C, 53.58; H, 3.79; N, 7.98; S, 9.20%.

*N-[2-(3,4-Dihydroxyphenyl)-4-oxo-thiazolidin-3-yl]-2-(7-{[2-(3,4-dihydroxyphenyl)-4-oxo-thiazolidin-3-ylcarbamoyl]-methoxy}-2-oxo-2H-chromen-4-yl)-acetamide* (**5e**). M.p. 198-200^◦^C, yield 47%; IR: ν_max_ 3,388 (NH), 2,922 (OH), 1,725, 1,694 (C=O, lactone), 1,619 (C=O, amide), 1,523, 1,444, 1,393, 1,284 and 1,152 cm^-1^; ^1^H-NMR: δ 11.98 (s, 1H, OH), 11.45 (s, 1H, OH), 8.41 (s, 1H, NH), 8.30 (s, 1H, NH-), 7.76 (d, 1H, H-5), 7.65-7.41 (m, 6H, arom.), 7.04 (d, 1H, H-6), 7.02 (s, 1H, H-8), 6.34 (s, 1H, H-3), 5.92 (s, 1H, NCHS), 4.77 (s, 2H, -OCH_2_), 4.22 (s, 2H, CH_2_), 3.38 (s, 2H, COCH_2_S); ^13^C-NMR: δ 35.7 (COCH_2_S), δ 45.5 (CH_2_), 57.4 (NCHS), 69.10 (CH_2_O-), 103.7 (C-3, from Ph), 107.6 (C-8), 111.0 (C-6), 112.5 (C-3), 113.4 (C-10), 115.4 (C-2, Ar-), 117.4 (C-5, Ar-), 122.2 (C-6, Ar-), 117.8 (C-5), 133.8 (C-1, Ar-), 143.0 (N=CH-), 147.4 (C-3, Ar-), 145.6 (C-4, Ar-), 151.2 (C-9), 155.0 (C-4), 160.3 (C-7), 160.9 (C-2), 166.4 (CONH-), 168.8 (COCH_2_S), 173.3 (CH_2_CONH-); Anal. Calcd. For C_31_H_26_N_4_O_11_S_2_: C, 53.60; H, 3.77; N, 8.07; S, 9.23; Found: C, 53.58; H, 3.79; N, 7.98; S, 9.20%.

*N-[2-(2,5-Dihydroxyphenyl)-4-oxo-thiazolidin-3-yl]-2-(7-{[2-(2,5-dihydroxyphenyl)-4-oxo-thiazolidin-3-ylcarbamoyl]-methoxy}-2-oxo-2H-chromen-4-yl)-acetamide* (**5f**). M.p. 221-223^◦^C, yield 46%; IR: ν_max_ 3,369 (OH), 3,286 (NH), 1,717, 1,681 (C=O, lactone), 1,667 (C=O, amide), 1,624 (C=C, arom.), 1,585, 1,492, 1,396, 1,267 and 1,156 cm^-1^; ^1^H-NMR: δ 11.95 (s, 1H, OH), 11.56 (s, 1H, OH), 8.48 (s, 1H, NH-), 8.34 (s, 1H, NH-), 7.76 (d, 1H, H-5), 7.68-7.30 (m, 6H, arom.), 7.04 (d, 1H, H-6), 7.02 (s, 1H, H-8), 6.34 (s, 1H, H-3), 5.92 (s, 1H, NCHS), 4.82 (s, 2H, -OCH_2_), 4.24 (s, 2H, CH_2_), 3.38 (s, 2H, COCH_2_S); ^13^C-NMR: δ 35.7 (COCH_2_S), δ 45.5 (CH_2_), 47.4 (NCHS), 69.1 (CH_2_O-), 107.6 (C-8), 111.0 (C-6), 112.5 (C-3), 113.4 (C-10), 115.4 (C-6, Ar-), 117.4 (C-3, Ar-), 115.6 (C-4, Ar-), 119.6 (C-1, Ar-), 127.8 (C-5), 143.0 (N=CH-), 148.7 (C-2, Ar-), 151.2 (C-9), 151.2 (C-5, Ar-), 155.0 (C-4), 160.2 (C-7), 160.9 (C-2), 166.4 (CONH-), 168.8 (COCH_2_S), 173.3 (CONH-); Anal. Calcd. For C_31_H_26_N_4_O_11_S_2_: C, 53.60; H, 3.77; N, 8.07; S, 9.23; Found: C, 53.58; H, 3.79; N, 7.98; S, 9.20%.

*N-[2-(4-Hydroxy-3-methoxyphenyl)-4-oxo-thiazolidin-3-yl]-2-(7-{[2-(4-hydroxy-3-methoxyphenyl-4-oxo-thiazolidin-3-ylcarbamoyl]-methoxy}-2-oxo-2H-chromen-4-yl)-acetamide* (**5g**). M.p. 217-218^◦^C, yield 84%; IR: ν_max_ 3,434, 3,224 (NH), 1,711, 1,671 (C=O, lactone), 1,632 (C=O, amide), 1,603 (C=C, arom.), 1,529, 1,394, 1,272 and 1,164 cm^-1^; ^1^H-NMR: δ 11.96 (s, 1H, OH), 8.19 (s, 1H, NH-), 8.10 (s, 1H, NH-), 7.77 (d, 1H, H-5), 7.40-7.21 (m, 6H, arom.), 7.04 (d, 1H, H-6), 7.02 (s, 1H, H-8), 6.34 (s, 1H, H-3), 5.92 (s, 1H, NCHS), 4.78 (s, 2H, -OCH_2_), 4.24 (s, 2H, CH_2_), 3.80 (s, 6H, -OCH_3_), 3.38 (s, 2H, COCH_2_S); ^13^C-NMR: δ 35.7 (COCH_2_S), δ 45.5 (CH_2_), 56.2 (OCH_3_), 57.8 (NCHS), 69.1 (CH_2_O-), 107.6 (C-8), 111.0 (C-6), 112.5 (C-3), 113.4 (C-10), 114.8 (C-2, Ar-), 117.0 (C-5, Ar-), 122.9 (C-6, Ar-), 132.4 (C-1, Ar-), 144.1 (C-4, Ar-), 151.2 (C-9), 151.5 (C-3, Ar-), 155.0 (C-4), 160.3 (C-7), 160.9 (C-2), 166.4 (CONH-), 168.8 (COCH_2_S), 173.0 (CONH-), Anal. Calcd. For C_33_H_30_N_4_O_11_S_2_: C, 54.84; H, 4.18; N, 7.75; S, 8.87; Found: C, 54.79; H, 4.19; N, 7.71; S, 8.82%.

*2-(2-Oxo-7-{*[4-oxo-2-(3-phenoxyphenyl)-thiazolidin-3-ylcarbamoyl]*-methoxy}-2H-chromen-4-yl)-N-[4-oxo-2-(3-phenoxyphenyl)-thiazolidin-3-yl]-acetamide* (**5h**). M.p. 221-222^◦^C, yield 57%; IR: ν_max_ 3,389, 3,071 (NH), 1,726, 1,685 (C=O, lactone), 1,628 (C=O, amide), 1,614 (C=C, arom.), 1,577, 1,490, 1,394, 1,261 and 1,156 cm^-1^; ^1^H-NMR: δ 8.30 (s, 1H, NH-), 8.21 (s, 1H, NH-), 7.76 (d, 1H, H-5), 7.70-7.10 (m, 18H, arom.), 7.04 (d, 1H, H-6), 7.02 (s, 1H, H-8), 6.34 (s, 1H, H-3), 5.92 (s, 1H, NCHS), 4.79 (s, 2H, -OCH_2_), 4.18 (s, 2H, CH_2_), 3.38 (s, 2H, COCH_2_S); ^13^C-NMR: δ 35.7 (COCH_2_S), δ 45.5 (CH_2_), 56.2 (OCH_3_), 57.6 (NCHS), 69.10 (CH_2_O-), 107.6 (C-8), 111.0 (C-6), 112.5 (C-3), 113.4 (C-10), 115.4 (C-4, Ar-), 116.1 (C-2, Ar-), 117.5 (C -2,6, Ar- PhO), 121.4 (C-4, Ar- PhO), 121.9 (C-6, Ar-), 127.8 (C-5), 128.5 (C-3,5 Ar- PhO), 128.6 (C-5 Ar-), 139.1 (C-1 Ar-), 151.2 (C-9), 155.0 (C-4), 156.8 (C-3, Ar-), 157.6 (C-1, Ar-PhO), 160.3 (C-7), 160.9 (C-2), 166.4 (CONH-), 168.9 (COCH_2_S), 173.3 (CH_2_CONH-); Anal. Calcd. For C_43_H_34_N_4_O_9_S_2_: C, 63.38; H, 4.21; N, 6.88; S, 7.87; Found: C, 63.34; H, 4.19; N, 6.86; S, 7.84%.

*N-*[2-(4-N,N-Dimethylaminophenyl)-4-oxo-thiazolidin-3-yl]*-2-(7-{[2-(4-N,N-dimethylaminophenyl)-4-oxo-thiazolidin-3-ylcarbamoyl]-methoxy}-2-oxo-2H-chromen-4-yl)-acetamide* (**5i).** M.p. 198-201^◦^C, yield 71%; IR: ν_max _3,398, 3,082 (NH), 1,724, 1,679 (C=O, lactone), 1,623 (C=O, amide), 1,604 (C=C, arom.), 1,554, 1,525, 1,364, 1,269 and 1,181 cm^-1^; ^1^H-NMR: δ 8.49 (s, 1H, NH-), 8.44 (s, 1H, NH-), 7.66 (d, 1H, H-5), 7.24-7.52 (m, 8H, arom.), 7.04 (d, 1H, H-6), 7.02 (s, 1H, H-8), 6.34 (s,1H,H-3), 5.92 (s, 1H, NCHS), 4.74 (s, 2H, -OCH_2_), 4.18 (s, 2H, CH_2_), 3.38 (s, 2H, COCH_2_S), 3.32 (s, 6H, -N(CH_3_)_2_), 2.99 (s, 6H, -N(CH_3_)_2_); ^13^C-NMR: δ 35.7 (COCH_2_S), δ 40.3 (CH_3_N-), 45.5 (CH_2_), 57.6 (NCHS), 69.10 (CH_2_O-), 107.6 (C-8), 111.0 (C-6), 112.5 (C-3), 113.4 (C-10), 114.4 (C-3,5, Ar-), 127.8 (C-5), 128.9 (C-1, Ar-), 130.1 (C-2,6 Ar-), 148.2 (C-4, Ar-), 151.2 (C-9), 155.0 (C-4), 160.3 (C-7), 160.9 (C-2), 166.4 (CONH-), 168.9 (COCH_2_S), 173.3 (CH_2_CONH-); Anal. Calcd. For C_35_H_36_N_6_O_7_S_2_: C, 58.64; H, 5.06; N, 11.72; S, 8.95; Found: C, 58.60; H, 4.98; N, 11.70; S, 8.90%.

*N-[2-(2-Hydroxy-5-nitrophenyl)-4-oxo-thiazolidin-3-yl]-2-(4-{[2-(2-hydroxy-5-nitrophenyl)-4-oxo-thiazolidin-3-ylcarbamoyl]-methyl}-2-oxo-2H-chromen-7-yloxy)-acetamide* (**5j**). M.p. 240-242^◦^C, yield 82%; IR: ν_max_ 3,367, 3,272 (NH), 1,689 (C=O), 1,618 (C=O, amide), 1,598 (C=C, arom.), 1,577, 1,517, 1,481, 1,342, 1,287 and 1,150 cm^-1^; ^1^H-NMR: δ 12.02 (s, 2H, OH), 8.71 (s, 1H, NH-), 8.59 (s, 1H, NH-), 7.67 (d, 1H, H-5), 7.32-7.54 (m, 6H, arom.), 7.04 (d, 1H, H-6), 7.02 (s, 1H, H-8), 6.34 (s, 1H, H-3), 5.92 (s, 1H, NCHS), 4.84 (s, 2H, -OCH_2_), 4.08 (s, 2H, CH_2_), 3.38 (s, 2H, COCH_2_S); ^13^C- NMR: δ 35.7 (COCH_2_S), δ 45.5 (CH_2_), 47.6 (NCHS), 69.10 (CH_2_O-), 107.6 (C-8), 111.0 (C-6), 112.5 (C-3), 113.4 (C-10), 116.9 (C-3, Ar-), 119.4 (C-1, Ar-), 121.8 (C-4, Ar-), 125.5 (C-6, Ar-), 127.8 (C-5), 141.1 (C-5, Ar-), 151.2 (C-9), 155.0 (C-4) 160.3 (C-7), 160.9 (C-2), 163.2 (C-2 Ar-), 166.4 (CONH-), 168.9 (COCH_2_S), 173.3 (CH_2_CONH-); Anal. Calcd. For C_31_H_24_N_6_O_13_S_2_: C, 49.47; H, 3.21; N, 11.17; S, 8.52; Found: C, 49.45; H, 3.19; N, 11.12; S, 8.50%.

*2-{2-Oxo-7-[(4-oxo-2-styrylthiazolidin-3-ylcarbamoyl)-methoxy]-2H-chromen-4-yl}-N-(4-oxo-2-styrylthiazolidin-3-yl)-acetamide* (**5k**). M.p. 221-224^◦^C, yield 48%; IR: ν_max_ 3,424, 3,276 (NH), 1,718 (C=O, lactone), 1,628 (C=O, amide), 1,613 (C=C, arom.), 1,560, 1,509, 1,393, 1,266 and 1,151 cm^-1^; ^1^H-NMR: δ 8.38 (s, 1H, NH-), 8.24 (s, 1H, NH-), 7.78 (2d, 4H, -HC=CH-), 7.64 (d, 1H, H-5), 7.04 (d, 1H, H-6), 7.02 (s, 1H, H-8), 6.34 (s, 1H, H-3), 5.92 (s, 1H, NCHS), 4.77 (s, 2H, -OCH_2_), 4.08 (s, 2H, CH_2_), 3.38 (s, 2H, COCH_2_S); ^13^C-NMR: δ 36.3 (COCH_2_S), δ 45.5 (CH_2_), 56.9 (NCHS), 69.10 (CH_2_O-), 107.6 (C-8), 111.0 (C-6), 112.5 (C-3), 113.4 (C-10) 123.8 (C-1, ethenyl-Ar)), 126.4 (C-2,6, Ar-), 128.0 (C-4, Ar-), 128.7 (C-3,5, Ar-), 129.6 (C-2, ethenyl-Ar), 135.2 (C-1, Ar-), 137.3 (N=CH-), 139.0 (C-3 Ar-), 151.2 (C-9), 155.0 (C-4), 160.2 (C-7), 160.9 (C-2), 166.4 (CONH-), 168.9 (COCH_2_S), 173.3 (CH_2_CONH-); Anal. Calcd. For C_35 _H_30 _N_4 _O_7_S_2_: C, 61.57; H, 4.43; N, 8.21; S, 9.39;Found: C, 61.56; H, 4.41; N, 8.19; S, 9.40%.
